# A Cross-Sectional Study of the Prevalence of Irritable Bowel Syndrome and Its Association With Anxiety and Depression Among Nurses in the Al-Qassim Region of Saudi Arabia

**DOI:** 10.7759/cureus.70278

**Published:** 2024-09-26

**Authors:** Abdullah N Alqifari, Shikhah G Alharbi, Fatimah M Alayed, Norah H Alabdullatif, Salman A Aljardan, Hana N Alqifari

**Affiliations:** 1 Department of Psychiatry, Qassim University, Buraydah, SAU; 2 College of Medicine, Qassim University, Buraydah, SAU; 3 Department of Statistics and Operation Research, College of Science, Qassim University, Buraydah, SAU

**Keywords:** a cross-sectional study, dass-21 scale, depression, ibs (irritable bowel syndrome), ocd/ anxiety disorders, saudi nurse

## Abstract

Introduction

Irritable bowel syndrome (IBS) is a prevalent functional gastrointestinal disorder frequently associated with psychological conditions such as stress, anxiety, and depression. Given the high-stress work environments of nurses and the lack of studies on this topic in the Al-Qassim region, this cross-sectional study aimed to investigate the prevalence of IBS and its association with anxiety and depression among nurses in this area of Saudi Arabia. The research hypothesis was that nurses with IBS would report higher levels of anxiety and depression compared to those without IBS.

Methods

This cross-sectional study was conducted among 96 nurses working at King Fahd Specialty Hospital (KFSH) in the Al-Qassim region. Nurses were selected using systematic sampling, with a total sample size initially calculated to be 189, but only 96 participated. Data were collected using a validated self-administered questionnaire, which included sociodemographic information and psychological assessments based on the Rome III criteria for IBS diagnosis and the Depression, Anxiety, and Stress Scales-21 (DASS-21) to measure psychological distress. Descriptive and inferential statistics were applied, with significance set at p < 0.05.

Results

In a sample of 96 nurses, the prevalence of IBS was found to be 19.8%, indicating a notable presence within this population. The majority of participants were female. No significant associations were observed between IBS and demographic or lifestyle factors. However, a higher prevalence of IBS was noted among nurses with more than 10 years of experience (27.3%, n=21) compared to those with less experience (10.5%, n=two), although this difference did not reach statistical significance (p=0.126). This study also revealed a high prevalence of stress, anxiety, and depression in the nurse population. Moreover, nurses diagnosed with IBS reported significantly higher levels of stress, depression, and anxiety compared to their counterparts without IBS (all p-values < 0.05).

Conclusion

There was a high prevalence of IBS among nurses, particularly those with higher levels of psychological distress. This study highlights the importance of developing targeted mental health interventions for nurses. However, the small sample size and cross-sectional design limit the generalizability and causality of the findings. Future studies should include larger, more diverse samples, and longitudinal designs to further explore these associations.

## Introduction

Irritable bowel syndrome (IBS) is a prevalent functional bowel illness linked to decreased quality of life and high healthcare expenses [1]. IBS affects approximately 9.2% of people globally, according to a meta-analysis of 53 studies utilizing the Rome III criteria across 38 countries, encompassing 395,385 participants [2]. IBS is characterized by altered bowel patterns and abdominal pain without intrinsic damage [3]. In addition to the motility issue, IBS is linked to several gastrointestinal and extraintestinal symptoms [3]. The mechanism underlying IBS remains unknown. However, there is evidence to support the idea that inflammatory and immunological variables, as well as disruption of brain-gut connections, are important contributors to disease manifestation [4,5]. IBS development, symptom intensity, and health outcomes are significantly affected by psychiatric diseases such as anxiety and depression [6]. Research has shown that there are physical changes that suggest impaired autonomic regulation, particularly in females with IBS and mood disorders, as well as those with a previous history of abuse [7].

Medical professionals, particularly nurses, are consistently exposed to elevated stress levels in their work environments, potentially increasing their risk of developing IBS [8]. Given the critical role of nurses in patient care and their frequent exposure to challenging working conditions, maintaining their optimal health and well-being is of paramount importance [9]. According to a study conducted in the United States (US), nurses have a higher-than-average risk of IBS due to working shifts that possibly affect circadian synchronization [10]. In Saudi Arabia, a cross-sectional study conducted at King Abdulaziz University Hospital (KAUH) in Jeddah, found that 33 out of 229 nurses were diagnosed with IBS, indicating a prevalence rate of 14.4%. Additionally, compared with the 9.3% prevalence of IBS among healthy nurses, nurses with significant anxiety had a prevalence of 24.5%. In contrast to the 13.2% prevalence among nurses without depression, the prevalence was 43.8% among nurses with severe depression [11]. A study conducted in Dammam, Saudi Arabia, found that up to 45.5% of nurses working in primary and secondary healthcare expressed work-related stress [12].

The association between irritable bowel syndrome and psychiatric disorders is well documented [13]. It is widely reported that there is a link between psychiatric diseases and gastrointestinal (GI) issues such as IBS [13]. Numerous clinical studies and reports found that 70-90% of IBS patients may also have psychiatric comorbidities, with mood and anxiety disorders being the most frequent [14]. IBS patients encounter difficulties in their social and professional lives and experience embarrassment regarding their symptoms. They often alter their dietary routines and turn to healthcare facilities in a fruitless effort to find proper medical care [15]. The high-stress environments nurses work in, combined with the physical and emotional demands of the profession, may increase their susceptibility to IBS and associated psychological conditions.

However, no study has specifically examined the prevalence of IBS and its relationship with anxiety and depression among nurses in the Al-Qassim region of Saudi Arabia. This research gap highlights the need for focused studies to better understand the mental and physical health challenges faced by nurses in this area. The primary aim of this study is to investigate the prevalence of IBS and its association with anxiety and depression among nurses in the Al-Qassim region. By exploring these associations, the study seeks to shed light on the mental and physical health burdens faced by nurses in high-stress work environments. Additionally, the study aims to contribute to the growing body of evidence on the brain-gut axis, particularly in the context of healthcare workers, and to identify potential areas for targeted interventions to improve both GI and psychological health in this vulnerable population. This study will help fill the existing research gap in the region and provide a foundation for future studies on IBS and mental health in nurses. The findings can also inform healthcare institutions about the importance of mental health support programs and the need for screening and managing IBS in their staff to enhance their well-being and overall job performance.

## Materials and methods

This cross-sectional study was conducted at King Fahd Specialty Hospital (KFSH) in the Al-Qassim region between June 2023 to April 2024. The inclusion criteria encompassed all nurses at KFSH, while those with organic gastrointestinal diseases were excluded.

Sample size

The required sample size for this study was calculated using Cochran’s formula (n = Z²P(1-P)/d²), which is commonly used to estimate sample sizes for proportions in large populations. Based on a 95% confidence level (Z = 1.96), a 5% margin of error (d = 0.05), and an estimated IBS prevalence of 14.4% (P = 0.144) from a previous study conducted among nurses in Jeddah​[11], the formula yielded a sample size of 189 participants. However, due to practical challenges in recruitment, including heavy workloads and limited availability of nurses during the data collection period, the final number of participants was 96. While this smaller sample size still allowed for meaningful statistical analysis, it may have limited the generalizability of the results.

Ethical consideration

Strict ethical protocols were adhered to, including obtaining written informed consent from all participants and securing approval from the King Fahd Specialty Hospital Research Committee (Approval No. 607/45/6180).

Questionnaire

Data were collected using a validated, self-administered questionnaire, which included sections on sociodemographic information (age, gender, nationality), professional background (specialty, years of experience), and lifestyle factors (exercise frequency, fast food consumption) (see Appendices). IBS was diagnosed using the Rome III criteria, which classifies functional gastrointestinal disorders. Psychological distress, including anxiety, depression, and stress, was measured using the Depression, Anxiety, and Stress Scales-21 (DASS-21).

Data cleaning and coding

Data cleaning involved a meticulous review to identify and correct any inconsistencies or missing values. Responses with incomplete answers were excluded to ensure the accuracy of the analysis. The data were then coded for statistical analysis, with each variable assigned a specific code. Two authors (ANA and NHA) independently verified the coding process to minimize errors and ensure accuracy. This dual-check process enhanced the reliability of the data analysis and the robustness of the study findings.

Statistical analysis

Descriptive statistics were utilized to summarize the data and identify patterns, whereas inferential statistics were applied to test hypotheses concerning the relationships between variables, thus ensuring a thorough analysis of the study's findings.

## Results

**Figure 1:**
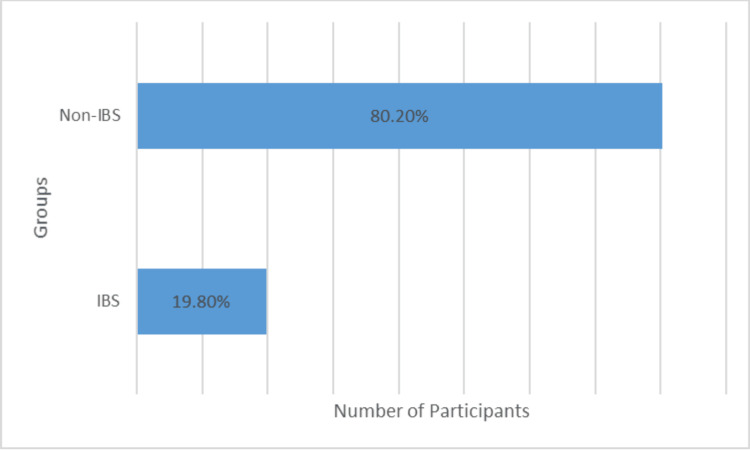
Prevalence of irritable bowel syndrome (IBS) among nurses working in the Al-Qassim region of Saudi Arabia

Nearly one in five nurses (19.8%, n=19) in our regional sample (N=96) reported experiencing IBS (Figure 1). We found no significant relationships between IBS among nurses and factors such as age (p=0.868), sex (p=0.505), nationality (p=0.419), years as a nurse (p=0.126), exercise frequency (p=0.152), or dietary fat intake (p=0.141) (Table 1). However, we observed a higher proportion of IBS among nurses with more than 10 years of experience (27.3%, n=21) than among those with less experience (10.5%, n=two), although this difference was not statistically significant (p=0.126).

**Table 1:** Relationship between sociodemographic factors and lifestyle, and IBS among nurses in the Al-Qassim region of Saudi Arabia (N=96) *significant at p<0.05 level. p-value was calculated using the likelihood ratio test IBS: irritable bowel syndrome

Variable		Count (%)		Likelihood ratio	p-value
		IBS n=19 (19.8%)	Non-IBS n=77 (80.2%)		
Age	≤ 35	16 (84.2%)	66 (85.7%)	0.028	0.868
	>35	3 (15.8%)	11 (14.3%)		
Gender	Male	0 (0%)	1 (1.3%)	0.444	0.505
	Female	19 (100%)	76 (98.7%)		
Nationality	Saudi	2 (10.5%)	4 (5.2%)	0.653	0.419
	Non-Saudi	17 (89.5%)	73 (94.8%)		
Years working as a nurse	≤10	17 (89.5%)	56 (72.7%)	2.346	0.126
	>10 years	2 (10.5%)	21 (27.3%)		
Exercise (days per week)	Never	9 (47.4%)	43 (55.8%)	6.710	0.152
	Once	8 (31.6%)	8 (10.4%)		
	2-3 times	1 (5.3%)	13 (16.9%)		
	>3 times	2 (10.5%)	6 (7.8%)		
	Daily	1 (5.3%)	7 (9.1%)		
Fat food consumption (in one week)	Once	12 (63.2%)	58 (75.3%)	5.460	0.141
	2-3 times	2 (10.5%)	14 (18.2%)		
	4-5 times	4 (21.1%)	4 (5.2%)		
	Daily	1 (5.3%)	1 (1.3%)		

**Figure 2:**
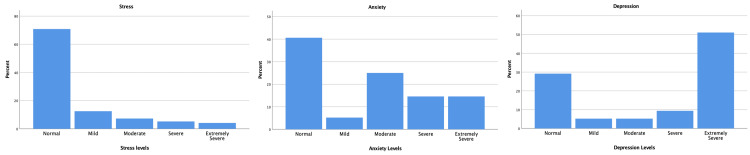
Self-reported prevalence of anxiety, stress, and depression among nurses Each bar chart represents levels of severity (e.g., normal, moderate, severe, and extremely severe) on the y-axis, with the corresponding percentage of nurses experiencing that level on the x-axis.

The self-reported prevalence of stress, anxiety, and depression among the nurses in our regional sample (N=96) was analyzed and categorized by severity (Figure 2). Stress emerged as a notable concern, with 3.84% (n=four) of nurses reporting extremely severe stress. Additionally, 25.33% (n=24) reported mild, moderate, or severe stress, while 70.83% (n=68) reported normal stress levels (p=0.005). Depression was also prevalent, with 51.04% (n=49) of nurses reporting extremely severe symptoms, and 19.79% (n=19) experiencing mild, moderate, or severe depression (p=0.004). Anxiety affected 14.58% (n=14) of nurses severely and another 14.58% (n=14) extremely severely. Moderate anxiety was reported by 25% (n=24) of the nurses, while 40.63% (n=39) reported normal levels of anxiety (p=0.028).

Nurses with IBS experienced a significantly higher prevalence of severe mental health symptoms compared to those without IBS (Table 2 and Table 3). Notably, 73.7% (n=14) of nurses with IBS reported experiencing extremely severe depression, compared to 45.5% (n=35) of nurses without IBS (p=0.004). Additionally, significantly fewer nurses with IBS reported experiencing normal stress levels (36.8%, n=seven) compared to their non-IBS counterparts (79.2%, n=six; p=0.005). The prevalence of extremely severe anxiety was also higher among nurses with IBS (36.8%, n=seven) compared to those without IBS (9.1%, n=seven; p=0.028). These findings highlight the significant impact of IBS on nurses' mental well-being and underscore the need for targeted interventions to support this population. Nurses with IBS also reported significantly higher mean scores for stress (18.21 vs. 9.43; p=0.004), anxiety (17.89 vs. 9.40; p=0.002), and depression (19.05 vs. 8.49; p<0.001) compared to those without IBS.

**Table 2:** The association between IBS and stress, anxiety, and depression among a sample of nurses (N=96) in the Al-Qassim region of Saudi Arabia. It reveals statistically significant relationships (p-value < 0.05) for all three factors *significant at p<0.05 level. p-value was calculated using the likelihood ratio test IBS: irritable bowel syndrome

Variable		IBS	Non-IBS	Likelihood ratio	p-value
		Count (%)			
Stress	Normal	7 (36.8%)	61 (79.2%)	15.07	0.005*
	Mild	5 (26.3%)	7 (9.1%)		
	Moderate	3 (15.8%)	4 (5.2%)		
	Severe	1 (5.3%)	4 (5.2%)		
	Extremely severe	3 (15.8%)	1 (1.3%)		
Anxiety	Normal	3 (15.8%)	36 (46.8%)	10.8	0.028*
	Mild	1 (5.3%)	4 (5.2%)		
	Moderate	5 (26.3%)	19 (24.7%)		
	Severe	3 (15.8%)	11 (14.3%)		
	Extremely severe	7 (36.8%)	7 (9.1%)		
Depression	Normal	0 (0%)	28 (36.4%)	15.6	0.004*
	Mild	1 (5.3%)	4 (5.2%)		
	Moderate	2 (10.5%)	3 (3.9%)		
	Severe	2 (10.5%)	7 (9.1%)		
	Extremely severe	14 (73.7%)	35 (45.5%)		

**Table 3:** The association between IBS and stress, anxiety, and depression among a sample of nurses (N=96) in the Al-Qassim region of Saudi Arabia using a two-sample independent t-test *significant at p<0.05 level. p-value was calculated using the likelihood ratio test IBS: irritable bowel syndrome

Score		Mean	Standard deviation	T	df	p-value
Stress score	Non-IBS	9.43	8.878	-3.212	24.020	0.004*
	IBS	18.21	11.07			
Anxiety score	Non-IBS	9.40	8.748	-3.361	25.047	0.002*
	IBS	17.89	10.121			
Depression score	Non -IBS	8.49	8.073	-4.190	23.827	<0.00*
	IBS	19.05	10.228			

## Discussion

To the best of our knowledge, this is the first study to examine the prevalence of IBS and its correlation with stress, anxiety, and depression among nurses in the Al-Qassim region. In this study, the prevalence of IBS was 19.8%, which is comparable to the reported 17.4% in Beijing, China [16] and slightly higher than the reported 14.4% in Jeddah, Saudi Arabia [11]. However, it is notably lower than the 28.0% prevalence reported in Seoul, Korea [8], particularly among female nurses, with a prevalence of 37.5% [17]. These variations may be attributed to differences in cultural backgrounds, ethnic groups, dietary habits, and workloads. Our findings align with the reported prevalence of IBS among the general population in Saudi Arabia, which is 20%, with a slightly higher rate of 22% among healthcare workers [18].

The prevalence of depression among nurses in this study was alarmingly high, with 71% exhibiting symptoms of depression, 51% of which were extremely severe. This significantly exceeds the prevalence of depression in the general Saudi Arabian population (12.7%) [19] and among migrant workers in Al-Qassim (20%), highlighting the unique stressors faced by this occupational group, particularly non-Saudi nurses, who constituted the majority of the study sample. Elevated rates of depression among nurses are not unique to this study, with comparable findings reported internationally: 32.4% in Australia [20], 37.2% in Japan [17], and 61.7% in China [16], almost similar to our study.

In our study, the prevalence of severe (14%) and extremely severe (15%) anxiety among nurses was notable, with moderate anxiety affecting 25% of nurses, while 41% reported normal levels. These findings are consistent with those of other similar studies. A study in Australia found that 41.2% of nurses scored above the normal threshold for anxiety [20], while a study in China reported that the prevalence of high anxiety (morbid anxiety) was 21.7% [16], and anxiety affected 70.7% of Egyptian healthcare workers [21]. Notably, the anxiety levels observed among nurses exceed those found in the general Saudi Arabian population, where 12.4% are at risk of generalized anxiety disorder (GAD) [19]. The prevalence of stress in our study was 29%, notably lower than findings from other studies. For example, a study in Australia found that 41.2% of participants exceeded the normal threshold for stress [20], while an Egyptian study reported a 56.9% prevalence of emotional stress among nurses [21].

Interestingly, no significant associations were found between IBS and demographic factors such as age, gender, and nationality, which contrasts with findings from studies in Western populations, where IBS is more prevalent in women [3]. One potential reason for the lack of a gender association in our study could be the relatively small sample size of male nurses, as also noted in similar studies conducted in Jeddah [11] and China [16]. Additionally, cultural and regional factors such as dietary patterns, social stressors, and healthcare environments may influence the gender distribution of IBS, though these factors were not deeply explored in this study. Future research should investigate the role of these cultural and regional differences in more depth, particularly in relation to the gender-based expression of IBS symptoms. The study by Ibrahim et al. [11] reported similar rates of IBS (13% vs. 15.6%) in nurses with more or less than 16 years of experience, aligning with our finding of no significant association between IBS and work experience. Additionally, our findings regarding the association between physical activity and IBS align with those of a Korean study that also found no significant association [22].

A strong association was found in our study between IBS and mental health, with nurses who have IBS displaying a higher prevalence of severe mental health symptoms compared to those without IBS. This was evident in the higher prevalence of extremely severe depression (73.7% vs. 45.5%), extremely severe anxiety (36.8% vs. 9.1%), and a lower reporting of normal stress levels (36.8% vs. 79.2%). This association between IBS and mental health aligns with a growing body of research. In general, our findings of a higher prevalence of anxiety and depression among IBS patients in this Middle Eastern social and cultural context are consistent with Asian and Western studies, as well as other Middle Eastern research [11,21,22]. Notably, a Chinese study found a greater prevalence of depression in IBS patients (31.4%) compared to a control group (17.5%). Furthermore, a community study revealed that IBS patients frequently experience anxiety, which often precedes the onset of IBS and significantly contributes to its development [16]. Moreover, a study in Korea showed that female nurses who had IBS were 2.214 times more likely to experience high-stress levels than those who did not have IBS [23]. It was also found that those with IBS were more likely to experience moderate to severe depressive symptoms compared to healthy controls. Given the demanding nature of nursing and its association with mental health symptoms, strategies to reduce these symptoms in nurses with IBS are urgently needed [23].

This study has several limitations that should be acknowledged. First, the small sample size, particularly the low number of male participants, limits the generalizability of the findings, particularly with regard to gender associations. Larger studies with a more balanced gender representation are needed to explore potential sex differences in IBS prevalence and its psychological comorbidities. Additionally, the study's cross-sectional design limits the ability to establish causality between IBS and psychological factors such as stress, anxiety, and depression. While associations were observed, it remains unclear whether these mental health issues contribute to the development of IBS or if IBS exacerbates psychological distress. Longitudinal studies are necessary to determine the directionality of these relationships.

Another limitation is the reliance on self-reported measures for both IBS diagnosis and psychological assessments. Although the Rome III criteria and DASS-21 scales are validated tools, self-reporting can introduce bias, including underreporting or overreporting of symptoms due to social desirability or stigma. Future studies may benefit from incorporating clinical diagnoses of IBS and objective mental health evaluations to strengthen the reliability of the findings. Furthermore, this study did not extensively explore lifestyle factors such as dietary habits, sleep patterns, or shift work, all of which could significantly influence both IBS and psychological well-being. The omission of these variables limits the comprehensiveness of the analysis. Future research should aim to integrate these factors to provide a more holistic understanding of the interplay between mental health, lifestyle, and gastrointestinal health in nurses. Lastly, the study was conducted in a single region of Saudi Arabia, which may limit the generalizability of the findings to other regions or countries with different cultural, environmental, and healthcare contexts. Multicenter studies across diverse geographical locations would help in confirming the findings and exploring regional variations in IBS prevalence and its association with mental health.

## Conclusions

This study revealed a high prevalence of IBS among nurses at KFSH. Nurses with IBS reported significantly higher levels of anxiety, depression, and stress compared to those without IBS. These findings highlight the critical need for targeted mental health interventions and support systems within nursing communities. Implementing well-being programs that provide mental health resources and dedicated time for self-care can mitigate the impact of IBS on nurses' mental health. Future research should focus on developing and evaluating specific interventions, such as stress management programs and mental health screening protocols, tailored for nurses at high risk of IBS. Additionally, longitudinal studies could help assess the long-term effects of these interventions and explore other contributing factors to IBS, including shift work and diet. Addressing these issues can ultimately enhance nurses' well-being and improve the overall healthcare environment. The insights from this study can inform clinical practices, guiding healthcare institutions to prioritize mental health care for their nursing staff and to integrate comprehensive support systems that promote both physical and mental well-being.

## Figures and Tables

**Figure 3:**
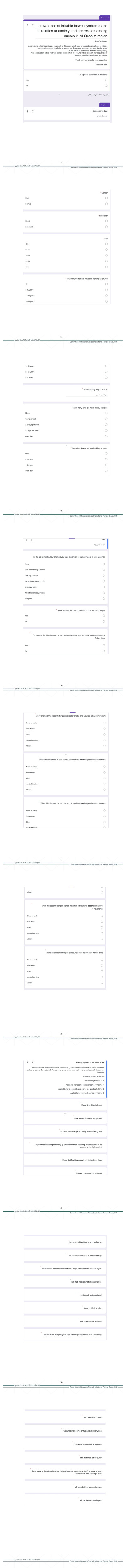
The questionnaire used in the study
